# A Cold Chain-Independent Specimen Collection and Transport Medium Improves Diagnostic Sensitivity and Minimizes Biosafety Challenges of COVID-19 Molecular Diagnosis

**DOI:** 10.1128/Spectrum.01108-21

**Published:** 2021-12-08

**Authors:** Vikram Saini, Priya Kalra, Manish Sharma, Chhavi Rai, Vikas Saini, Kamini Gautam, Sankar Bhattacharya, Shailendra Mani, Kanchan Saini, Sunil Kumar

**Affiliations:** a Laboratory of Infection Biology and Translational Research, Department of Biotechnology, All India Institute of Medical Sciences, New Delhi, India; b Biosafety Laboratory-3, Centralized Core Research Facility (CCRF), All India Institute of Medical Sciences (AIIMS), New Delhi, India; c Defence Institute of Physiology and Allied Sciences (DIPAS), Defence Research and Development Organization (DRDO), Ministry of Defense, Delhi, India; d University College of Medical Sciences and Guru Teg Bahadur Hospital, New Delhi, India; e Translational Health Science and Technology Institute (THSTI), Faridabad, Haryana, India; University of Georgia

**Keywords:** biosafety, cold chain, COVID-19 testing, diagnostics, SARS-CoV-2, VTM, virology, qRT-PCR

## Abstract

Equitable and timely access to COVID-19-related care has emerged as a major challenge, especially in developing and low-income countries. In India, ∼65% of the population lives in villages where infrastructural constraints limit the access to molecular diagnostics of COVID-19 infection. Especially, the requirement of a cold chain transport for sustained sample integrity and associated biosafety challenges pose major bottlenecks to the equitable access. Here, we developed an innovative clinical specimen collection medium, named SupraSens microbial transport medium (SSTM). SSTM allowed a cold chain-independent transport at a wide temperature range (15°C to 40°C) and directly inactivated SARS-CoV-2 (<15 min). Evaluation of SSTM compared to commercial viral transport medium (VTM) in field studies (*n* = 181 patients) highlighted that, for the samples from same patients, SSTM could capture more symptomatic (∼26.67%, 4/15) and asymptomatic (52.63%, 10/19) COVID-19 patients. Compared to VTM, SSTM yielded significantly lower quantitative PCR (qPCR) threshold cycle (*Ct*) values (mean Δ*Ct* > −3.50), thereby improving diagnostic sensitivity of SSTM (18.79% [34/181]) versus that of VTM (11.05% [20/181]). Overall, SSTM had detection of COVID-19 patients 70% higher than that of VTM. Since the logistical and infrastructural constraints are not unique to India, our study highlights the invaluable global utility of SSTM as a key to accurately identify those infected and control COVID-19 transmission. Taken together, our data provide a strong justification to the adoption of SSTM for sample collection and transport during the pandemic.

**IMPORTANCE** Approximately forty-four percent of the global population lives in villages, including 59% in Africa (https://unhabitat.org/World%20Cities%20Report%202020). The fast-evolving nature of SARS-CoV-2 and its extremely contagious nature warrant early and accurate COVID-19 diagnostics across rural and urban population as a key to prevent viral transmission. Unfortunately, lack of adequate infrastructure, including the availability of biosafety-compliant facilities and an end-to-end cold chain availability for COVID-19 molecular diagnosis, limits the accessibility of testing in these countries. Here, we fulfill this urgent unmet need by developing a sample collection and transport medium, SSTM, that does not require cold chain, neutralizes the virus quickly, and maintains the sample integrity at broad temperature range without compromising sensitivity. Further, we observed that use of SSTM in field studies during pandemic improved the diagnostic sensitivity, thereby establishing the feasibility of molecular testing even in the infrastructural constraints of remote, hilly, or rural communities in India and elsewhere.

## INTRODUCTION

Despite a remarkably fast development of vaccines and their success in reducing the severity of infections in several countries, the extremely contagious nature and evolution of immune escape variants of SARS-CoV-2 necessitate adoption of “Test, Track, Treat and Vaccinate” strategy for controlling transmission of COVID-19 infection. Quickly and accurately identifying those infected and isolating them has successfully worked globally as a major disease containment and management strategy (1). Molecular testing method of quantitative real-time PCR (qRT-PCR) remains the cornerstone and the “gold standard” for accurate diagnosis of SARS-CoV-2 due to its high sensitivity and specificity (2) and familiarity with the technique even in low- and middle-income countries. Nonetheless, it becomes extremely difficult to use qRT-PCR-based molecular diagnosis in a highly populous, geographically large and diverse setting like India, which had been reeling with a “tsunami” of COVID-19 infections. In April to June 2021, India had frequent days of over 400,000 new infections per day, the highest in the world. During this period, 1 in every 3 deaths reported worldwide each day due to COVID-19 was reported in India. The contagion has spread to rural areas and tier 2 and 3 cities in India where infrastructural and technical constraints particularly limit the access to diagnosis and consequent health care. Therefore, innovative and quick solutions are needed to control COVID-19 spread.

Major challenges (Fig. S1) to the accessibility of molecular diagnostic services to the masses include (i) a requirement of an end-to-end maintained cold chain for the transport of clinical samples, (ii) biosafety considerations of transporting an infectious/suspected infectious biological sample in a virus transport medium (VTM), wherein virus remains viable until it is taken to the lab at a low temperature (2 to 8°C), (iii) requirement of a biosafety cabinet to ensure a safe and WHO-compliant molecular diagnostic testing, and (iv) loss or reduction in sensitivity of molecular diagnosis due to deterioration of clinical sample quality/stability owing to various factors, like sample complexity, glitches with cold chain maintenance, degradation of nucleic acids, or delay in sample processing (3–5). In particular, maintenance of a cold chain throughout the process, until the sample goes for RNA isolation, and the requirement of a class II biosafety cabinet are difficult to achieve in smaller cities/towns, villages, and remote areas due to the infrastructural constraints and limited resources (6). Last, the transport of a biological contagious material requires special packing and handling of samples while wearing personal protective equipment (PPE), thereby increasing assay cost and limiting affordability.

In this study, we address these challenges head-on by developing a clinical specimen collection and transport medium, which we name SupraSens microbial transport medium (SSTM), that is compatible with the qRT-PCR-based molecular diagnostics. Using cell culture assays and field studies in patient cohorts, we show that SSTM allows cold chain-independent transport of samples at ambient temperature, directly inactivates SARS-CoV-2 in the specimen within 15 min, and improves RNA recovery, thereby improving the overall sensitivity of diagnosis. Evaluation of SSTM under field conditions vis-a-vis commercial viral transport medium (*n* = 181 patients) revealed that SSTM usage yielded significantly reduced *Ct* values in the specimens of same patients, indicating a better sample RNA recovery from samples handled in SSTM. This reduction in *Ct* values manifests in a significant improvement in the diagnostic sensitivity (70%) compared to that of the traditional VTM, which showed only 58.82% sensitivity in our studies. Considering that the logistical and infrastructural constraints, including cold chain, biosafety, and loss of sensitivity, are not unique to India, our study highlights the invaluable global utility of this SSTM for molecular diagnosis of COVID-19, especially during the pandemic.

## RESULTS

### SSTM has robust microbicidal activity, including against SARS-CoV-2 virus.

**FIG 1:**
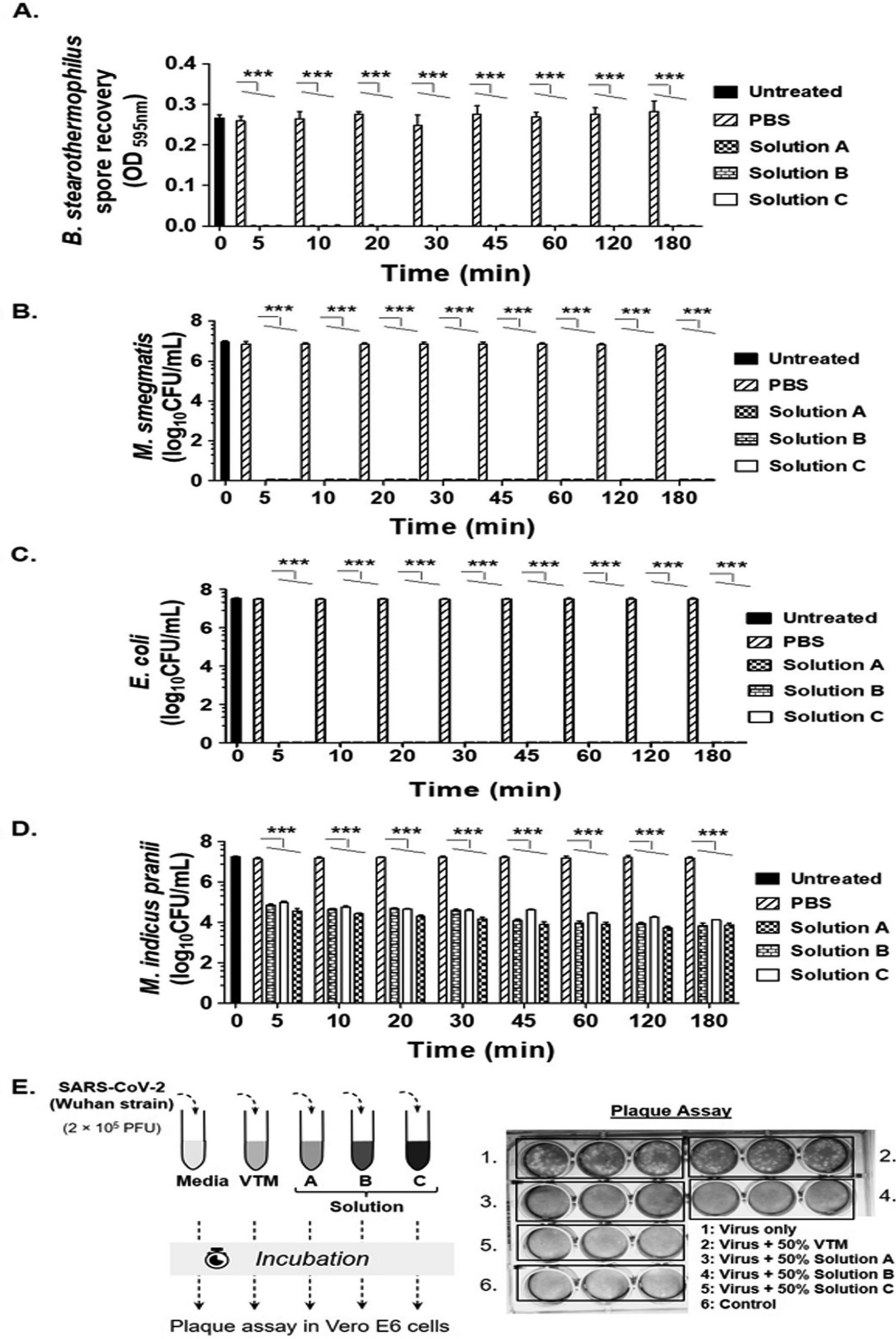
Microbicidal potential of various in-house formulations of microbial transport media. Various microbes, namely, (A) *B. stearothermophilus* spores, (B) M. smegmatis, (C) E. coli, (D) *M. indicus pranii*, and (E) SARS-CoV-2 (Wuhan strain), were incubated in different formulations (solutions A, B, C) of our in-house microbial transport medium (MTM) to test microbe inactivation. Microbe recovery (A to D) was compared as PBS versus treatment groups (solutions A, B, C) using a two-way ANOVA Tukey multiple-comparison test (***, *P* < 0.0001 for PBS versus solution A, B, or C; panels A to D). Presence/absence of recovered SARS-CoV-2 plaques was scored visually (E). Data represent mean ± standard deviation of experiments performed at least thrice in duplicates. Untreated group represents starting inoculum of bacilli/spores and PBS-treated group as control.

The traditional viral transport media (VTM) known to date are designed to preserve and maintain viruses within clinical samples in a viable state. However, for a molecular PCR-based diagnosis method, virus viability is not required. Rather, an optimal transport medium for molecular diagnosis method should be able to reduce the biosafety risk by minimizing, inactivating, or neutralizing microbe without compromising diagnostic sensitivity of assay. Based on this premise, we developed different formulations of microbial transport media (MTM), namely, solutions A, B, and C. We observed that following exposure with all three solutions, microorganisms, namely, Bacillus stearothermophilus spores (Fig. 1A), Mycobacterium smegmatis (Fig. 1B), and Escherichia coli (Fig. 1C), were completely inactivated at each time point (exposure time 5 min to 180 min) and showed no recovery, as opposed to respective control (phosphate-buffered saline [PBS])-exposed cultures. Moreover, all the solutions could reduce the viable Mycobacterium indicus pranii bacilli by 2 to 3 log_10_ with increasing time, with solution A showing maximum bactericidal effect at early time points (Fig. 1D). Likewise, with M13KE bacteriophage, the highest reduction (∼3-fold) was also observed with solution A (Fig. S2).

We next evaluated the virucidal potential of the solutions in question. Indeed, unlike that with VTM, incubation with even 50% diluted solution A, B, or C for 15 min inactivated SARS-CoV-2 virus, as is evident by the absence of any plaques in the corresponding wells compared to the control (incubation with complete medium) (Fig. 1E). Similar results were obtained when the virus was incubated for 60 min (data not shown). In summary, our formulations could successfully inactivate a broad range of microbes, including SARS-CoV-2 virus. Solution A demonstrated maximum broad spectrum microbicidal activity and hence was pursued for further studies. We use the terminology SupraSens MTM (SSTM) for solution A henceforth.

### SSTM improves RNA recovery from human cells at room temperature and above.

**FIG 2:**
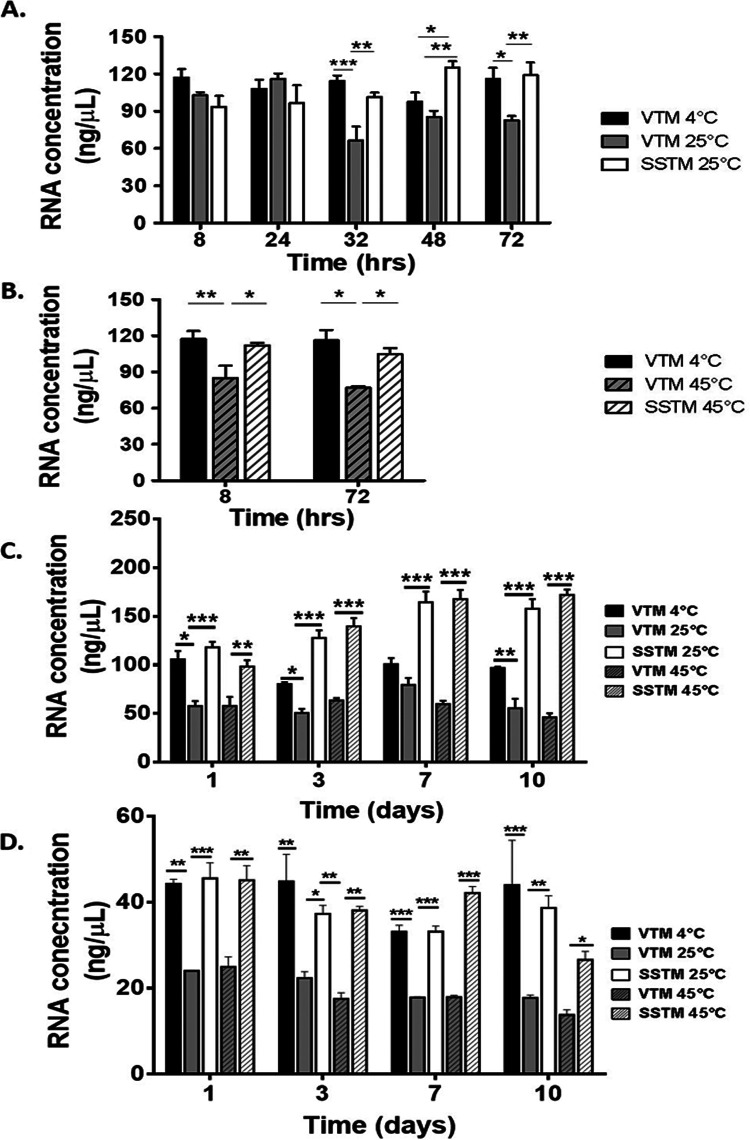
SSTM yields a significantly improved RNA recovery in a time- and temperature-dependent manner. (A) RNA recovered from HEK293 cells exposed to SSTM or VTM at 25°C. (B) RNA recovery from HEK293 cells at 45°C. For both A and B, RNA was isolated by using automatic nucleic acids extraction system (Promega Maxwell automated system, USA), and RNA recovery from commercial VTM at 4°C was used as a control. (C) RNA recovery by a manual extraction method using standard TRIzol reagent followed by DNase treatment. (D) RNA recovery by a manual extraction method using total RNA purification kit (Norgen Biotek Corp., Canada). Both C and D capture comprehensive time point analysis of RNA recovery from the HEK293 cells at day 1, day 3, day 7, and day 10. The significance of differences in RNA recovery in VTM versus SSTM at each temperature and time point was ascertained by using two-way ANOVA Tukey’s multiple-comparison test (A and B) or one-way ANOVA Tukey’s multiple-comparison test (C and D) (*, *P* < 0.05; **, *P* < 0.001; ***, *P* < 0.0001 are significant). Data represent mean ± standard error of the mean (SEM) performed at least twice in duplicates.

Next, we evaluated the utility of SSTM in molecular diagnostic testing in terms of sample nucleic acid recovery. Considering the ongoing pandemic situations and complex nature of clinical samples, we first decided to evaluate utility of SSTM in a laboratory cell culture set up in terms of recovery and stability of nucleic acids at two temperatures (25°C and 45°C) and compare it with the RNA recovery in commercial VTM in standard sample transport conditions of COVID-19 diagnostics (i.e., 4°C). Using a human embryonic kidney cell line (HEK293) as a surrogate for host cells, we showed that SSTM yields an RNA recovery significantly higher (Fig. 2) than that of commercial VTM at 25°C (ambient temperature) and 45°C, the high temperature that could prevail in large parts of India during summers. Furthermore, we noticed that the RNA recovery with SSTM at 25°C and 45°C is comparable to the RNA recovery from commercial VTM at standard cold chain temperature (4°C). Importantly, RNA recovery from commercial VTM was compromised in both a time- and temperature-dependent manner. At 25°C in commercial VTM, RNA recovery dropped past 24 h, and by 72 h, we observed significant reduction in RNA recovery compared to that in commercial VTM (4°C) and SSTM (25°C) (Fig. 2A). This decline in performance of commercial VTM vis-a-vis RNA recovery manifested as early as 8 h when temperature increased to 45°C (Fig. 2B). These observations are independent of our starting cell numbers, as a similar decline in RNA recovery is also observed even at lower cell numbers (5 × 10^4^) (data not shown). The data clearly suggested the excellent potential of SSTM for RNA recovery and stability compared to commercial VTM even in the absence of a cold chain. Further, for all the samples, the 260/280 ratio was consistently in the range of 1.8 to 2.0, indicating a high level of purity of recovered RNA. Nonetheless, the possibility of DNA contamination could not be completely ruled out.

To ensure that the effect on RNA recovery is independent of RNA extraction kit used, we adopted two more methods of RNA recovery, including the standard TRIzol method followed by DNase treatment (Fig. 2C) and use of a total RNA purification kit (Norgen Biotek Corp., Canada) (Fig. 2D). We observed a similar effect on RNA recovery wherein there is a greater RNA recovery from SSTM compared to that from VTM, irrespective of the temperature and time, even when sample is processed as late at 10 days (Fig. 2C and D).

The results above as well as the excellent virucidal potential of SSTM shown earlier provided us the rationale to further evaluate utility of SSTM with actual clinical samples.

### Utility of SSTM with clinical samples: compatibility and suitability with downstream qRT-PCR COVID-19 diagnostics.

Being cognizant of the greater complexity of a clinical sample compared to that of a traditional cell culture setup, we first evaluated the clinical utility of SSTM. First, we determined whether SSTM can be used to process the clinical samples without altering the workflow of diagnosis (Fig. S3A) and accurately capture COVID-19 positives and negatives as identified by VTM in qRT-PCR methodology. We observed that SSTM was compatible with the diagnostics workflow and could correctly score all confirmed positives (10/10) and confirmed negatives (5/5) identified using VTM workflow. Testing of additional symptomatic patient samples (*n* = 15) further identified 4 patients as positive when processed with VTM. A parallel processing of those same samples with SSTM, however, identified 4 additional samples as positive (increase of 26.6%, *n* = 15) for COVID-19 infection, which were designated negative based on the *Ct* value cutoffs. We noticed that SSTM yielded lower *Ct* values captured in qPCR assays (mean reduction in Δ*Ct* of 1.75 ± 0.63, 95% confidence interval [CI] of −3.75 to 0.25) compared to VTM. Our findings conclusively established that samples undergoing SSTM treatment remain amenable to downstream analysis during clinical molecular diagnostics without any compromise in assay sensitivity. These experiments therefore provided a strong justification to evaluate the performance of SSTM as a viable medium for clinical sample collection and transport and to compare its overall diagnostic performance compared to that of commercial VTM (Fig. S3B).

### Application of SSTM for sample collection and transport at field temperature reveals a significantly higher sensitivity of COVID-19 diagnosis compared to that of VTM on cold chain.

During the field studies, the samples were collected from 181 patients (142 male, 39 female; 16 years to 78 years; median age 39 years; Table S1). Out of these, 57 individuals were symptomatic, while the rest (124 individuals) were asymptomatic at the time of sample collection. We observed that samples collected and transported at cold chain in commercial VTM could diagnose only 20 positives (20/181; positivity rate = 11.05%), while for 3 samples the results were inconclusive (i.e., signal was detected only from a single viral gene; classified inconclusive as per the instructions for interpretation of clinical result from the FDA-approved respective RT-PCR kits). For the same patients, samples collected at the same time and transported at the prevailing field temperature using SSTM, we observed a significantly higher detection rate with 34 confirmed positive cases and 3 samples being inconclusive. The positivity rate with SSTM improved to 18.78% (34/181). Data analysis revealed that SSTM is not only able to detect all the positive samples identified by commercial VTM but also far exceeds the sensitivity of detection by capturing samples detected as inconclusive or negative when processed using commercial VTM. Out of these 34 patients diagnosed as infected with SARS-CoV-2, 15 were clinically symptomatic and 19 were asymptomatic contacts. Therefore, samples collected in VTM could capture only ∼73.33% (11/15) of symptomatic and 47.36% (9/19) of asymptomatic infected cases. The overall increase in SSTM sensitivity over that of VTM was a significant 70% (14/20). On the other hand, the reduction in sensitivity of qRT-PCR-based detection of COVID-19 attributed to change in VTM collection and transport medium was a remarkable 41.17% (14/34). Our studies clearly demonstrate the great utility of SSTM, as it not only did not require cold chain to transport samples but also could capture additional infected people, including those who are asymptomatic.

### Sample collection and transport in SSTM ensures better RNA recovery compared to VTM.

**FIG 3:**
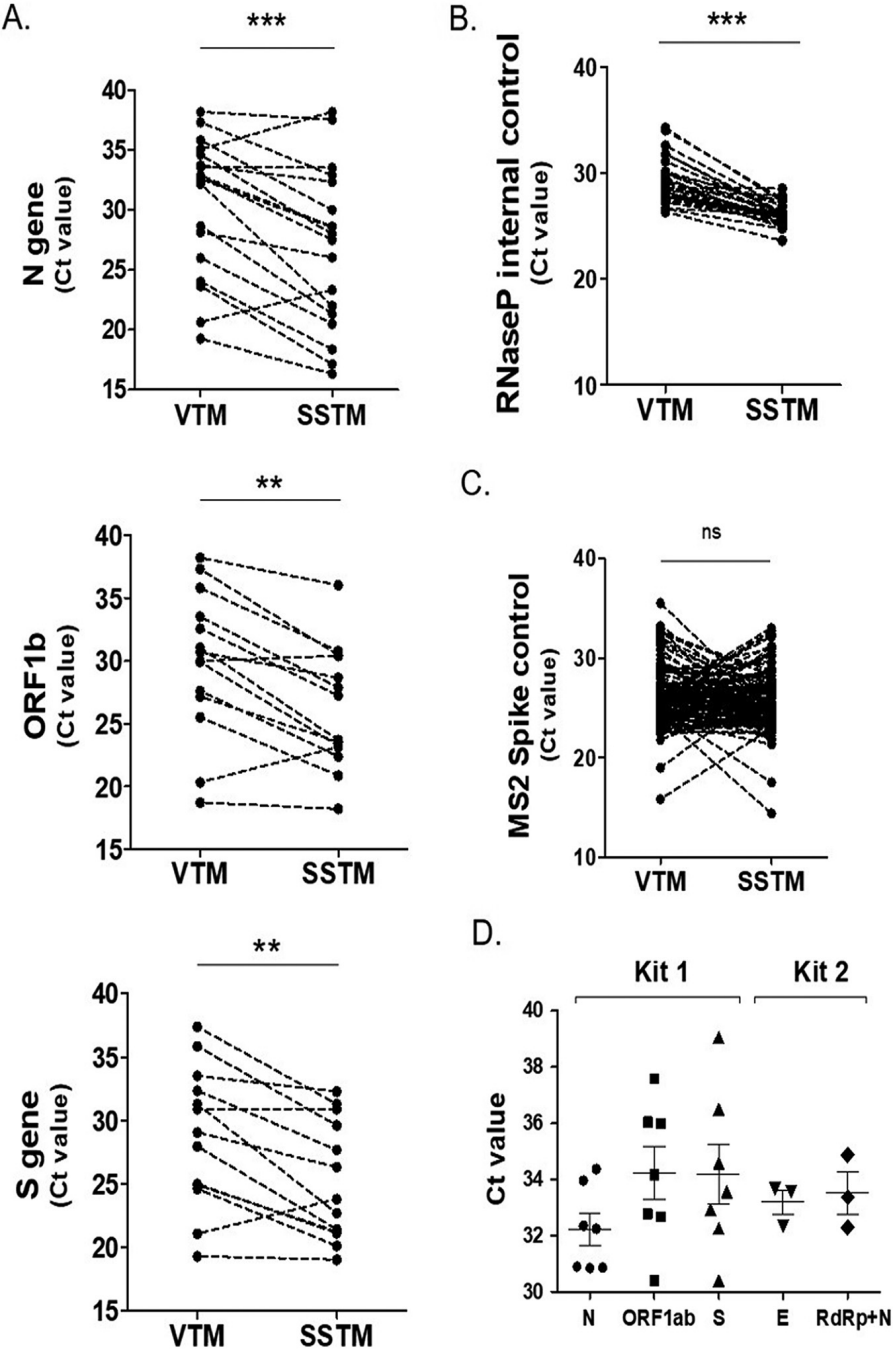
Distribution of qPCR *Ct* values of paired samples indicates improved RNA recovery with SSTM as sample collection and transport medium. Analysis of *Ct* values for COVID-19 genes. (A) N gene, ORF1ab, S gene using TaqPath COVID-19 qPCR kit. (B) Identical amounts of MS2 phage were added to all sample pairs prior to processing to compare efficiency of RNA extraction. Represented are MS2 qPCR *Ct* values for the same samples processed by VTM or SSTM (*n* = 136). (C) RNase P gene of humans was targeted as internal positive control for sample quality maintenance and process of qPCR validation (*n* = 31). (D) *Ct* value distribution of SARS-CoV-2 genes in asymptomatic individuals that were scored positive by SSTM but missed by VTM (by either detection kit used). Statistical significance was evaluated using paired *t* test (**, *P *< 0.001; ***, *P* < 0.0001; *P* > 0.05 is not significant [ns]). (Note: each dot represents individual qPCR *Ct* values. Dotted lines represent a paired match of values in VTM versus SSTM [panels A to C]. Solid lines in panel D represent mean ± standard error).

To investigate the likely basis of this improved COVID-19 diagnosis with SSTM, we speculated if a better nucleic acids recovery from clinical samples, as observed in our *in vitro* experiments, could contribute to improved detection rate by SSTM. A paired comparison of *Ct* values for individual viral genes from samples scored positive by both VTM and SSTM highlighted a clear and substantial reduction in *Ct* values for SSTM compared to those for VTM for the individual viral genes, namely, N gene (mean Δ*Ct* = −3.71; 95% CI = −5.442 to −1.982, *P* = 0.0003, *n* = 18), ORF1ab gene (mean Δ*Ct* = −3.70; 95% CI = −5.440 to −1.954, *P* = 0.0005, *n* = 14), and S gene (mean Δ*Ct* = −3.88; 95% CI = −5.41 to −1.61, *P* = 0.0034, *n* = 13) (Fig. 3A). The observation that the *Ct* values produced from commercial VTM were higher than those of the SSTM suggested a lower RNA recovery from the commercial VTM sample, especially because other possible confounding factors, like instruments and reagents, remained the same. Further analysis showed that when MS2 phage (spiked in samples) was used as an internal control (acts as a control for RNA extraction), we observed only a negligible change in MS2 *Ct* values (mean *Ct* in VTM of 25.68 versus *Ct* in SSTM of 25.10, 95% CI = −0.930 to 0.186; mean Δ*Ct* = −0.578, *P* = 0.183, *n* = 136) in SSTM with respect to those in VTM (Fig. 3B).

In another subset of our cohort, we used human RNase P as internal control, which not only acts as a control for RNA extraction and the amplification reaction but also assesses the quality of clinical samples. We observed a reduction in qPCR *Ct* value of RNase P gene when SSTM was used for sample collection (mean Δ*Ct* = −3.071; 95% CI = −3.7 to −2.43, *P* = 0.0001, *n* = 32; Fig. 3C). Compared to that of the spiked MS2 phage RNA, which acted as a control for the extraction and downstream processing once the sample was inside the lab, a significantly lower *Ct* value confirmed an improved stability and/or recovery of RNA when samples are collected in SSTM, which is consistent with results of our *in vitro* studies in the cell culture models (Fig. 2). This also indicates a critical impact of sample collection medium on maintenance of sample quality and a consequent impact on molecular diagnosis. These findings are also corroborated in our cohort (*n* = 32; median age = 42, males = 26, females = 6) wherein we used RNase P control and a different qPCR diagnostic kit (TRUPCR SARS-CoV-2 RT-qPCR kit). A mean reduction of −1.652 in Δ*Ct* values of RdRP+N gene (95% CI = −5.156 to 1.853, paired comparison of *n* = 6) was observed with SSTM, compared to those with VTM, confirming that these consistently lower *Ct* values with SSTM were independent of the qPCR diagnostic kit employed. A reduction in *Ct* values for various target genes for qRT-PCR kit where matched values were available, and scoring of additional positive patients by having within-range *Ct* values, strongly indicated a greater RNA recovery and increased sample stability in SSTM that accounts for the superior performance of SSTM over that of the commercial VTM in COVID-19 molecular diagnostics.

### Evaluation of sensitivity and specificity of SSTM and VTM highlights the superiority of SSTM in molecular diagnosis of COVID-19 infection.

As shown earlier in our study, use of SSTM allowed significantly higher additional detection of COVID-19-infected individuals compared to use of commercial VTM. We first argue that positive results identified in our study using SSTM are true positives. This is because the diagnostic probes of the qPCR kits used are highly specific to SARS-CoV-2 and simultaneously target multiple genes of the virus for specificity (7, 8). Therefore, the SSTM methodology can affect the quality and quantity of only specimen RNA and does not interfere with the reactions/probes or diagnostic reaction *per se*. Out of 14 additional diagnosed patients with SSTM, 4 were symptomatic and fully met the *Ct* value-based cutoff criterion (9). The remaining 10 were asymptomatic and were household contacts of confirmed COVID-19 patients. An analysis of *Ct* value for these asymptomatic patients showed that their qRT-PCR yielded signals for all the genes under consideration and produced appropriate amplification curve, ruling them out to be artifact, a well-established approach for accurate diagnostics and ruling out false positives (9, 10). Further, the *Ct* values for viral genes in these samples were well below the recommended threshold *Ct* values, supporting their categorization as true positive (for first kit [*n* = 7], N gene: mean *Ct* value = 32.21 ± 0.558, 95% CI = 30.85 to 33.58; ORF1ab gene: mean *Ct* value = 34.23 ± 0.935, 95% CI = 31.94 to 36.52; S gene: mean *Ct* value = 34.18 ± 1.082, 95% CI = 31.53 to 36.83) (for second kit [*n* = 3], RdRp+N gene: mean *Ct* value = 33.51 ± 0.748; E gene: mean *Ct* value = 33.19 ± 0.426) (Fig. 3D).

**FIG 4:**
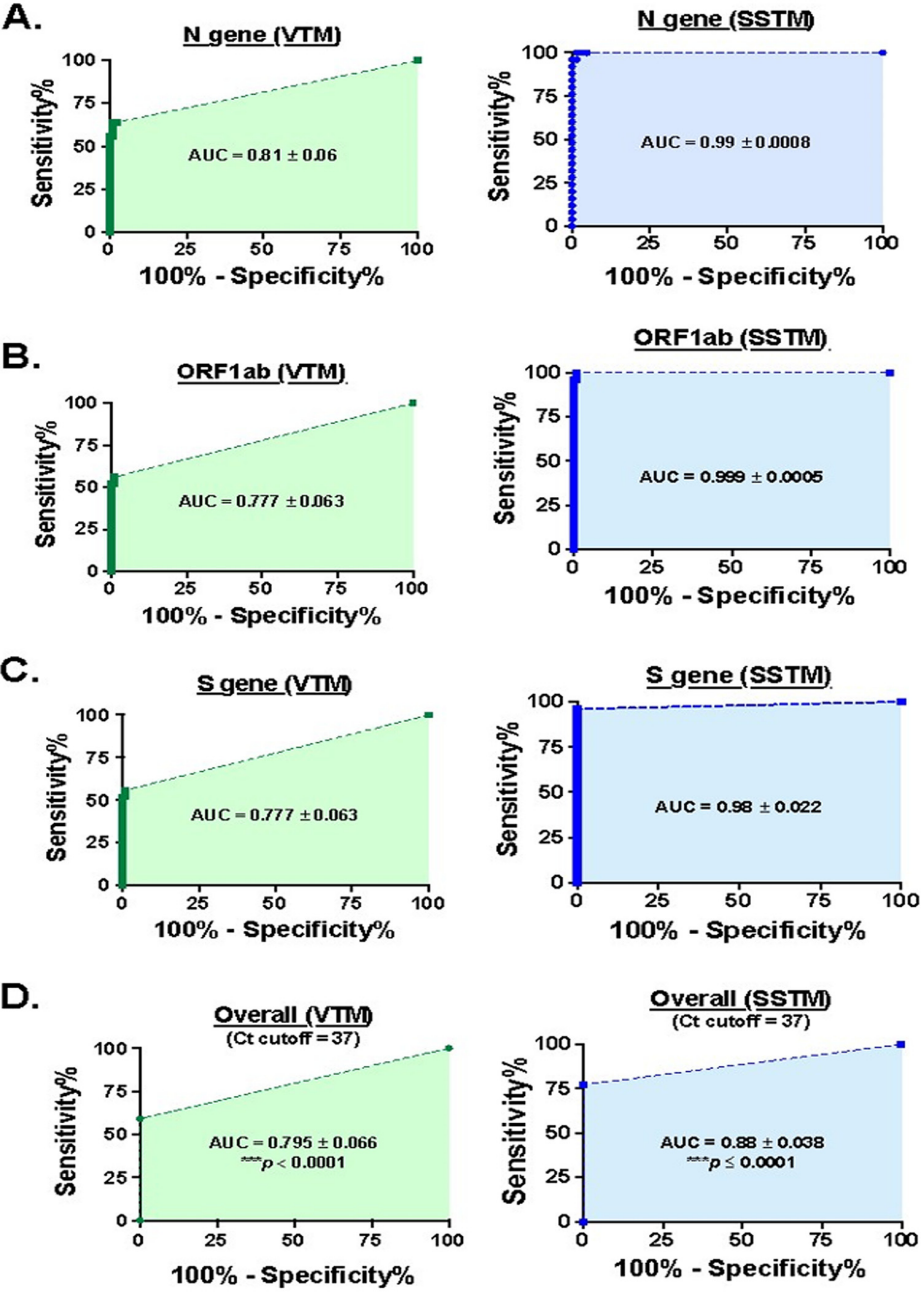
ROC curve analysis of clinical specimens’ qPCR data indicates a significant loss of sensitivity of COVID-19 detection in samples processed in VTM. ROC curves were generated using the qPCR *Ct* values of (A) N gene, (B) ORF1ab, and (C) S gene in VTM-processed samples, taking SSTM as reference. (D) Binary coding was assigned to each clinical specimen by combining the qPCR data of all three COVID-19 genes, and ROC curve analysis was performed. The diagnostic test performance was compared as ROC (VTM) versus ROC (SSTM) for each set, using 2-sample Z test (***, *P* < 0.0001). (Note: TaqPath COVID19 combo kit defines a *Ct* cutoff value of <37 for COVID-19 disease. Specimens where qPCR signals were “not detected” due to low/no viral load were assigned a *Ct* of 42, for ROC analysis).

Considering the increased sensitivity of detection with the usage of SSTM as sample collection and transport medium, we next used SSTM as the reference to compare the performance of VTM as a method of sample collection. Based on this premise, we first categorized the SSTM-identified-positive clinical specimens as “patient group” (*n* = 25) and the remaining 124 specimens as “control group” (total 149 samples processed with the FDA-approved TaqPath COVID-19 combo kit, Thermo Fischer Scientific). Thereafter, we performed the receiver operating characteristic (ROC) curve analysis, as it is a popular assessment tool to compare performance of diagnostic tests (11). We observed that VTM usage had a significantly lower efficiency of viral RNA detection, as substantiated by a much lower area under the curve (AUC) for individual viral genes N, ORF1ab, and S gene in VTM (0.81, 0.78, and 0.78) versus SSTM (0.99, 0.99, and 0.98), respectively (***, *P* < 0.0001; Fig. 4A to C).

Since TaqPath COVID-19 qPCR kit qualifies a sample to be positive only if two or more viral genes show *Ct* value of ≤37, we converted the *Ct* value data to binary coding (1 for *Ct* of ≤37 and 0 for *Ct* of >37) to obtain combined effect of transport medium on diagnosis efficiency. To determine whether binary conversion led to any loss of data, we evaluated the AUC of the ROC curves for the binary-coded data for all three genes used in the assay (Fig. S4). We observed a similarity between the AUC values of ROC curves of binary converted data versus those of original *Ct* values (Fig. 4 versus Fig. S4), confirming the suitability of our approach for ROC analysis by combining independent data points of all three genes. Following integration, as observed with individual genes, VTM method was found to have overall sensitivity (AUC = 0.78) significantly lower than that of SSTM method (AUC = 1.0; ***, *P* < 0.0001; Fig. 4D). Table 1 summarizes the observed diagnostic parameters (sensitivity, specificity, negative predictive value, positive predictive values) with use of VTM or SSTM as the sample transport medium. Overall, we observed no change in assay specificity with either reagent but did observe a remarkable >40% decline in the sensitivity with VTM compared to that of SSTM. Also, the negative predictive value (NPV) for VTM is lower than that of SSTM (91.9% versus 100%; Table 1), implying that VTM usage may miss some positive samples as false negatives. Last, we observed a higher sensitivity of detection of N gene compared to that of ORF1ab or S gene by either VTM or SSTM (Table 1). These results are also corroborated in a similar analysis of qPCR data (*n* = 32) obtained with the TRUPCR SARS-CoV-2 RT-qPCR kit, wherein we observed ∼33% loss in the sensitivity with VTM compared to that with SSTM (*, *P* = 0.0003) (Table S2).

**TABLE 1:** Evaluation of loss of COVID-19 diagnostic test performance^*a*^ in clinical samples processed with VTM versus SSTM

Diagnostic test and genes tested	VTM method (patients = 25; controls = 124)				SSTM method (patients = 25; controls = 124)				% loss in VTM sensitivity^*b*^	Z test comparing sensitivity % of VTM versus SSTM^*c*^	
	Sensitivity^*d*^ % (CI)	Specificity^*e*^ % (CI)	NPV^*f*^ % (CI)	PPV^*g*^ % (CI)	Sensitivity % (CI)	Specificity % (CI)	NPV % (CI)	PPV % (CI)		Z-score	*P* value
TaqPath COVID-19 Combo kit											
N gene	60.0 (40.7–76.6)^*h*^	99.2 (95.6–99.9)	92.5 (86.7–95.9)	93.8 (71.7–98.9)	100 (86.7–100)	98.4 (94.3–99.6)	100 (96.9–100)	92.6 (76.6–97.9)	40.0	8.6	0.00
ORF1ab	52.0 (33.5–70)	100 (97–100)	91.2 (85.2–94.9)	100 (77.2–100)	92.0 (75–97.8)	100 (97–100)	98.4 (94.4–99.6)	100 (87.5–100)	40.0	7.7	<0.0001
S gene	56.0 (37.1–73.3)	99.2 (95.6–99.9)	91.8 (85.9–95.4)	93.3 (70.2–98.8)	88.0 (70–95.8)	100 (97–100)	97.6 (93.3–99.2)	100 (85.1–100)	32.0	6.2	<0.0001
Overall kit result	56.0 (37.1–73.3)	100 (97–100)	91.9 (86–95.4)	100 (78.5–100)	100 86.7–100)	100 (97–100)	100 (97–100)	100 (86.7–100)	44.0	9.2	0.00

a COVID-19 diagnostic test performed using TaqPath COVID-19 combo kit (Thermo Fisher Scientific) (*n* = 149). Diagnostic test performance evaluated using online tool https://ebm-tools.knowledgetranslation.net/calculator/diagnostic (24, 25).

*^b^*% loss in VTM sensitivity = % sensitivity in SSTM − % sensitivity in VTM.

c Z test for comparison of sensitivities between VTM versus SSTM using online tool https://epitools.ausvet.com.au/ztesttwo (26, 27).

d sensitivity % = [true positive/(true positive + false negative)] × 100.

e specificity % = [true negative/(true negative + false positive)] × 100.

f negative predictive value (NPV) % = [true negative/(true negative + false negative)] × 100.

g positive predictive value (PPV) % = [true positive/(true positive + false positive)] × 100.

h Values in parentheses denote 95% confidence interval.

## DISCUSSION

Several countries, including India, have witnessed a tsunami of COVID-19 infections, causing a substantial loss of life and warranting urgent public health measures to halt the transmission and prevent new infections. With infection spreading to the hinterlands, villages, and small towns in India, which are home to ∼65% of population, the challenge to identify those infected early, especially those who are asymptomatic, and isolate them has become particularly acute. An absolute essentiality of a cold chain for sample transport, especially in remote and rural areas, a class II type biosafety cabinet, and last mile connectivity to a time-sensitive assay remains a major global challenge to the access of qRT-PCR-based molecular diagnostics, which remains the gold standard in the field (2, 5).

For a molecular PCR-based diagnosis, viral viability is not required. Rather, an optimal transport medium for molecular diagnosis method should be able to reduce the biosafety risk by minimizing viability/inactivating a microbe and maintain the sample quality. Our data show severe attenuation of a broad range of resilient microbes and a complete inactivation of SARS-CoV-2 within 15 min of exposure, thereby limiting the biological risk from a spill/breakage, a requirement of a biosafety-compliant transport. Especially in the context of COVID-19 infection, complete killing of SARS-CoV2 would eliminate or minimize the need to have a class II biosafety cabinet (12). These requirements constitute a major bottleneck in ramping up the diagnostics facilities in small towns and cities (Fig. S1). Furthermore, biosafety cabinets are difficult to transport and set up in remote rural and hilly areas that have limited road connectivity.

Another major challenge to equitable access of diagnostics services is the maintenance of cold chain, especially in the rural, hilly, and small towns. In these geographies, in addition to the limited resources, electricity supply is often erratic and disruptions are not uncommon. For this reason, we performed the evaluation of SSTM at 25°C and 45°C and not at 4°C, as the whole premise of developing a different VTM was to mitigate the requirement of cold chain. Data from our *in vitro* experiments and results of field trials clearly indicate that SSTM does not compromise the diagnosis efficiency or RNA recovery from the samples even at higher temperature. In fact, the meteorological record of Delhi (https://amssdelhi.gov.in/), the site of field studies, shows that temperature during the study period varied from 15°C to 40°C (mean temperature for minimum [24.3°C ± 5.35; 95% CI = 18.71°C to 29.95°C) and maximum temperature [35.33 ± 4.37°C; 30.75°C to 39.92°C]. These data clearly indicate the utility of SSTM at a broad range of temperatures, making it ideal not only for India but globally, especially for other low- to mid-income countries.

A crucial observation was that SSTM consistently showed lower *Ct* values for candidate viral genes in the patient samples. On average, SSTM-derived samples yielded a *Ct* reduction of ∼3.8 for N, ORF1ab, and S genes. It is known that a change/reduction in *Ct* value by 3.3 roughly translates into ∼10 times more target RNA, suggesting a better recovery of RNA that would facilitate better diagnostics. In our study, these differences in *Ct* values translated into a 70% increase in sensitivity for SSTM over that of samples processed in commercial VTM. In this study, commercial VTM could diagnose only ∼73.3% (11/15) of the symptomatic cases, while this performance dropped down to ∼47.40% (9/19) in cases of asymptomatic patients. Clearly, SSTM demonstrated a greater sensitivity not only with symptomatic patients but with the asymptomatic ones as well by picking one and a half times more samples than commercial VTM. Contribution of asymptomatic infections to COVID-19 spread and control is well documented, as even asymptomatic infected people are known to do viral shedding and spread infection (13–15).

Several important factors substantiate that the additional findings of COVID-19 infection by SSTM are true positives. First, our samples qualify the pretest probability assessment of COVID-19, including symptoms, any potential exposure to COVID-19, and likelihood of an alternative diagnosis (16). Out of 14 additional patients identified by SSTM as positive for SARS-CoV-2 infection, about 29% were symptomatic and ∼71% were the household contacts of confirmed positive patients. Further, our qRT-PCR diagnostic assays relied on detection of multiple viral genes specific only to SARS-CoV-2 and demonstrated *Ct* values well below the cutoff thresholds (9, 10). Importantly, for all the regions tested in our assays, including nucleocapsids (N), envelope (E), RNA polymerase-dependent RNA (RdRp), ORF1ab, and spike (S), we observed significantly lower *Ct* values in SSTM contributing to the improved sensitivity and detection. The improved sensitivity of SSTM and its ability to enhance RNA recovery could be especially useful to mitigate loss of sensitivity associated with sample pooling strategy. This strategy is gaining traction to improve throughput when infection prevalence comes down while eliminating ∼75% of testing reactions (17). Therefore, our findings are of special importance, as they improve the detection of both symptomatic and asymptomatic patients. Achieving higher sensitivity is especially critical as asymptomatic COVID-19 patients are a major contributory factor in community spread or transmission of SARS-CoV-2 (14, 15). In cases of symptomatic patients, missing the diagnosis using qRT-PCR has been a frequent issue in the hospital settings and chest computed tomography (CT) is advised as a supplemental diagnostic tool for such patients (18). This could be concerning, especially because CT has a lower specificity than RT-PCR testing (19). Also, CT scan is often not an accessible service in smaller towns and villages, and the patient has to travel, risking exposure of SARS-CoV-2 to the providers. This missed diagnosis by qRT-PCR is often attributed to low viral load or the mutation in the target viral genes, especially in the spike region that renders primer binding inefficient (9, 10) (https://www.fda.gov/medical-devices/letters-health-care-providers/genetic-variants-sars-cov-2-may-lead-false-negative-results-molecular-tests-detection-sars-cov-2).

The present study thus brings attention to a critical but unappreciated aspect of COVID-19 diagnostics, i.e., role of sample collection and transport medium in sensitivity of diagnostics. Clinical sample is prone to quality loss and degradation due to presence of various other microflora and enzymes, including RNases. Any loss of cold chain or delay in processing, which often happens at the time of extensive workload and depleted personnel, would affect the diagnostic quality. Our observation of higher test positivity with SSTM than with VTM method clearly highlights that sample stability and a consequent improved RNA recovery in our collection and transport medium result in a reduction in *Ct* values with SSTM, which have a significant impact on improving the sensitivity of detection.

To summarize, SupraSens microbial transport medium, or SSTM, is compatible with molecular diagnostic testing platforms and offers features far superior to those of traditional VTM currently in use. Based on extensive *in vitro* studies and evaluation of SSTM in the operational settings in field, it is confirmed that unlike the traditional VTM, SSTM (i) is stable and can function at broad temperature range (eliminates a cold chain requirement), (ii) can directly inactivate a broad range of microbes, including SARS-CoV-2 (minimizes the biosafety concern of handling infectious sample), (iii) is compatible with COVID-19 diagnostics workflow, (iv) preserves nucleic acids integrity in samples and improves recovery (resulting in better diagnostic sensitivity), and (v) significantly improves SARS-CoV-2 detection even in asymptomatic individuals. Therefore, we strongly recommend adoption of SSTM to facilitate a last mile connectivity, enhanced detection of symptomatic and asymptomatic infections, and ensure availability of molecular diagnosis testing even in the remotest settings, as well as rural and semiurban settings. Since the logistical and infrastructural constraints, including cold chain, biosafety, and loss of sensitivity, are not unique to India, our study highlights the invaluable global utility of SSTM for sample collection and transport during the pandemic.

## MATERIALS AND METHODS

### Chemical reagents and laboratory strains.

Molecular biology-grade chemicals, such as Tris, sodium chloride (NaCl), EDTA, sodium bicarbonate, potassium chloride, disodium hydrogen phosphate, potassium dihydrogen phosphate, hydrochloric acid, etc., were used in this study. Various microbe media formulations used in this study were purchased from HiMedia Laboratories (India). Tissue culture-grade reagents, including trypsin, fetal bovine serum, and Dulbecco’s modified Eagle’s medium, were procured from Invitrogen (USA) and Sigma (USA). Various formulations evaluated in our study, including SupraSens microbial transport medium (SSTM), were developed in-house using molecular biology-grade reagents. SSTM is under patent consideration (Indian patent application no. 202011042597). Microorganisms tested in this study included the saprophytic, hygromycin B-resistant, recombinant laboratory strains of E. coli and M. smegmatis, M. indicus pranii, Bacillus stearothermophilus spores (Sigma-Aldrich, USA), and SARS-CoV-2 (Wuhan strain). HEK293T cell line was a kind gift from Shyam S. Chauhan, Department of Biochemistry, AIIMS, New Delhi, India. Various kits used for automatic RNA isolation were Maxwell RSC viral total nucleic acid purification system (Promega, USA), TRIzol-based RNA extraction (20), SARS-CoV-2 RNA extraction kit (PathKits, India), and total RNA purification kit (Norgen Biotek Corp., Canada). For qRT-PCR assays, we used approved kits by ICMR, India or FDA, USA for COVID-19 diagnostics and included Q-Line ER-nCoV-19 RT-PCR assay kit (Q-line, India), TaqPath COVID-19 combo kit (Thermo Fischer Scientific, USA), and TRUPCR SARS-CoV-2 RT-qPCR kit (v.3.2; 3B Blackbio Biotech India Ltd.). For VTM solution and swabs, we used the ICMR-approved VTM/swab set from HiMedia or PathKits India.

### Evaluation of microbial inactivation potential by various MTM formulations.

For this purpose, various molecular biology-grade reagents with varied concentrations were mixed and evaluated against different microbes to obtain an optimal and most-effective combination for microbial transport medium (MTM). We later refer to this MTM as SupraSens microbial transport medium, or SSTM, which would result in maximum attenuation in a broad range of microbes, including SARS-CoV-2. For initial screening, bacterial strains of E. coli, M. smegmatis, and *M. indicus pranii* were aerobically grown in Luria–Bertani (LB) broth or Middlebrook 7H9 broth (supplemented with 10% albumin-dextrose-NaCl complex), respectively, at 37°C, 180 rpm until log-phase (21, 22). Approximately 10^7^ CFU of respective bacteria were harvested, washed with 1× PBS, and either left untreated (in 500 μL of 1× PBS, 0 min time point) or treated with 500 μL of test MTM solutions (A, B, C) or 1× PBS for various time points (5, 10, 20, 30, 45, 60, 120, 180 min) at room temperature (RT). The bacterial recovery in MTM at each time point was compared with respect to the PBS-treated and untreated “0 min” samples by CFU plating of various dilutions of untreated/treated bacterial cultures on culture media as indicated previously (22).

We also used biological indicator strips coated with 10^6^ spores of *B. stearothermophilus* (Sigma-Aldrich, USA) as gold standard to confirm microbe neutralization/inactivation in our MTM formulations. Briefly, untreated or treated *B. stearothermophilus* spore strips of various time points were recovered in brain heart infusion (BHI) medium at 55°C, 180 rpm for 3 to 7 days, followed by comparison of optical density at 696 nm (OD_595_) at various time points as MTM-treated versus PBS-treated or untreated group. Development of turbidity was indicative of spore viability or failure of neutralization. Untreated group denoted the starting inoculum of bacilli, i.e., initial CFU in the sample, while the PBS-treatment group acted like a control that received a volume of PBS equivalent to that of MTM groups.

### Evaluation of SARS-CoV-2 inactivation potential of various MTM formulations.

To determine the utility of candidate MTMs against SARS-CoV-2, 150 μL of infected Vero-E6 culture supernatant containing approximately 2 × 10^5^ PFU of SARS-CoV-2 (Wuhan strain) was diluted in equal volume of complete growth medium (DMEM-2% fetal calf serum [FCS]), commercial VTM, or different candidate MTM formulations, namely, solution A, solution B, or solution C, for different time points (15 min or 60 min). Briefly, virus supernatants were diluted in the candidate MTMs (1:1), and within the minutes postdilution (15 min or 60 min), 100 μL of the respective mixes were serially diluted in DMEM-2% FCS and overlaid on Vero-E6 monolayer of cells to produce 30 to 40 plaques per well. After infection for 1 h at 37°C and 5% CO_2_, the inoculum was discarded, the monolayer was washed with incomplete DMEM, and 1 mL of DMEM-2% FCS supplemented with carboxy-methyl cellulose per well was overlaid on the cells. The plates were then incubated at 37°C and 5% CO_2_ for at least 72 h. Subsequently, the plates were then fixed with 4% paraformaldehyde and stained with 1% crystal violet in 20% methanol to score for the plaques.

### RNA isolation to study the effect of MTM on RNA recovery using cell lines.

For these experiments, we used HEK293 cells and seeded them at two densities, 1 × 10^5^ cells/0.3 mL or 5 × 10^4^ cells/0.3 mL, in replicate tubes containing either commercial VTM or solution A MTM (SupraSens or SSTM) and incubated at temperatures 4°C (VTM tube), 25°C (both VTM and SSTM tubes), and 45°C (both VTM and SSTM tubes) for various time periods (8 to 240 h). At designated time points, from each tube, 300 μL sample was removed for RNA isolation, either by automatic nucleic acids extraction (Maxwell RSC viral total nucleic acid purification system, Promega, USA) or manually using either TRIzol method followed by DNase treatment or total RNA purification kit (Norgen Biotek Corp., Canada). The RNA concentrations were thereafter quantified by Cytation 1 imaging multimode reader (Biotek, USA) for each sample and normalized against cell number. For each experiment, RNA purity for each sample was also ascertained by 260/280 ratio.

### Analysis of suitability of SSTM for downstream qRT-PCR screening of clinical samples for COVID-19.

Briefly, 30 precollected nasopharyngeal specimens (NPS) in 3 mL of commercial VTM (10 confirmed positive and 5 confirmed negative) were used in this step (23 male, 7 females; 9 years to 86 years; mean age: 45 years). Here, in addition to normal processing of samples for RNA extraction and qRT-PCR analysis, one additional aliquot of 300 μL of sample was also processed likewise, except that it was transferred to SSTM prior to RNA extraction and further downstream process. RNA was extracted using the Indian Council of Medical Research (ICMR, India)-approved coronavirus SARS-CoV-2 RNA extraction kit (PathKits, India). qRT-PCR assays for SARS-CoV-2 were performed using ICMR, India-approved Q-Line ER-nCoV-19 RT-PCR assay kit (Q-line, India) for COVID-19 diagnosis. Presence of virus was determined through the identification of two genetic markers: envelope (env or E) gene and RNA-dependent RNA polymerase (RdRP) gene. The kit also employs a human housekeeping gene as internal positive control (IPC) to ensure human sample availability and quality of nucleic acid for the reference of gene expression. The samples were reported as positive or negative based on the *Ct* values specified by the manufacturer.

### Evaluation of SSTM in the field settings in paired match with VTM.

Field studies to evaluate SSTM were facilitated by Defense Research and Development Organization’s COVID-19 diagnostics laboratory at Defense Institute of Physiology and Allied Sciences (DIPAS), New Delhi, India. The laboratory is approved by ICMR India for COVID-19 diagnostics and is accredited by NABL (National Accreditation Board for Testing and Calibration Laboratories, India) with its accreditation system established in accordance with ISO/IEC 17011. Briefly, the study evaluated 181 (142 male, 39 female; median age = 39; 57 symptomatic, 124 asymptomatic) matched VTM and SSTM samples (pairs) from individuals in either a health care or community setting collected by a trained team of paramedics. As per the study protocol, nasopharyngeal specimens (NPS) from suspected patients were collected using sterile flocked nylon swabs (HiMedia, India) by a team of trained health care workers. Two samples were simultaneously collected from each individual using swabs and immediately transferred in sterile tubes containing 3 mL of either VTM or SSTM. The VTM sample was cold chain transported (4°C) as per the diagnostic guidelines, while samples in SSTM were transported at prevailing field temperature without any cold chain. These samples were processed and tested at COVID-19 Diagnostics Centre of DRDO at DIPAS, New Delhi, India. Prior to processing, all samples were coded and all downstream processing personnel (sample collection team, molecular biology team, and data analysis and diagnosis team) were blinded to the sample identity. Sample RNA was isolated as per the manufacturer’s instructions of QIAmp mini viral RNA extraction kit (Qiagen, USA). For COVID-19 diagnostics, qRT-PCR was performed using FDA-approved kits, namely, TaqPath COVID-19 combo kit (Thermo Fischer Scientific) or TRUPCR SARS-CoV-2 RT-qPCR kit (v.3.2; 3B Blackbio Biotech India Ltd.) as per their availability during the pandemic. Importantly, samples in commercial VTM or SSTM for the same patient were always tested using same kit/machine to ensure fair comparison of performance. The manufacturer’s guidelines of the COVID-19 RT-PCR kits were used to interpret the qRT-PCR results (positive/negative) as per the existing ICMR guidelines. The TaqPath COVID-19 combo kit deems a tested sample positive for SARS-CoV2 if at least 2 out of 3 genes (N gene, ORF1ab, S gene) it tests demonstrate a qPCR *Ct* value of ≤37. Similarly, TRUPCR SARS-CoV-2 RT-qPCR kit deems a tested sample positive for SARS-CoV2 if at least 1 out of 2 gene combinations (E gene, RdRp+N gene) it tests demonstrates a qPCR *Ct* value of ≤35.

### Evaluation of diagnostic performance of SSTM and VTM.

The diagnostic performance of VTM versus SSTM methods in clinical sample pairs, including designated control group and COVID-19-positive patient group, was determined by a ROC curve analysis on qPCR *Ct* value of individual target gene using the modality in GraphPad PRISM (v7.0) software. For each gene, the values of area under the curve (AUC) were obtained from ROC plots to compare diagnostic test performance of VTM versus SSTM. Furthermore, since each type of COVID-19 qPCR kit assays multiple COVID-19 genes, to evaluate the overall kit performance, the *Ct* value data were converted into binary coding (23, 24). For example, specimens displaying a *Ct* value for a gene below or equal to the recommended cutoff were coded as 1, and specimens displaying a *Ct* value for a gene above the recommended cutoff were coded as 0. In each kit, the specimen data for all the screened genes were combined for patients as well as control samples and similarly analyzed by ROC curve method (described above) to compare the overall performance of VTM versus SSTM.

For the determination of sensitivity, specificity, negative predictive value (NPV), and positive predictive value (PPV) for all qPCR assays, we used a web-based diagnostic test calculator (https://ebm-tools.knowledgetranslation.net/calculator/diagnostic) (25, 26), a tool previously used for detection of sensitivity and specificity of qRT-PCR assay.

### Statistical analyses.

Statistical analyses were performed with the help of GraphPad PRISM software (v7.0). Unless specified, for microbicidal assay and RNA quantifications studies using cell line, we used two-way analysis of variance (ANOVA) with either Tukey’s or Sidak’s multiple-comparison test to evaluate the significance of observed differences. Analysis of differences in total RNA recovery using Norgen RNA isolation kit was performed by one-way ANOVA with Sidak’s multiple-comparison test. Differences in paired *Ct* values for qPCR internal control and SARS-CoV-2 genes were scored using paired *t* test. Statistical comparison of patient diagnostic parameters and ROC curves was performed by 2-sample Z test using online tool https://epitools.ausvet.com.au/ztesttwo (27, 28). In every statistical analysis, a *P* value of ≤0.05 was considered significant.
