# Supplementary material for: A Cold Chain-Independent Specimen Collection and Transport Medium Improves Diagnostic Sensitivity and Minimizes Biosafety Challenges of COVID-19 Molecular Diagnosis
Source: Microbiol Spectr. 2021 Dec 8;9(3):e01108-21. doi: 10.1128/Spectrum.01108-21 (PMC8653843; doi:10.1128/Spectrum.01108-21)
Supplement: SUPPLEMENTAL FILE 1 — Supplemental material. Download SPECTRUM01108-21_Supp_1_seq8.pdf, PDF file, 0.4 MB [file spectrum01108-21_supp_1_seq8.pdf]

SUPPLEMENTAL INFORMATION

SUPPLEMENTAL FIGURES

Supplemental Figure S1 Schematic representing various challenges in the Covid-19 molecular diagnostics workflow.

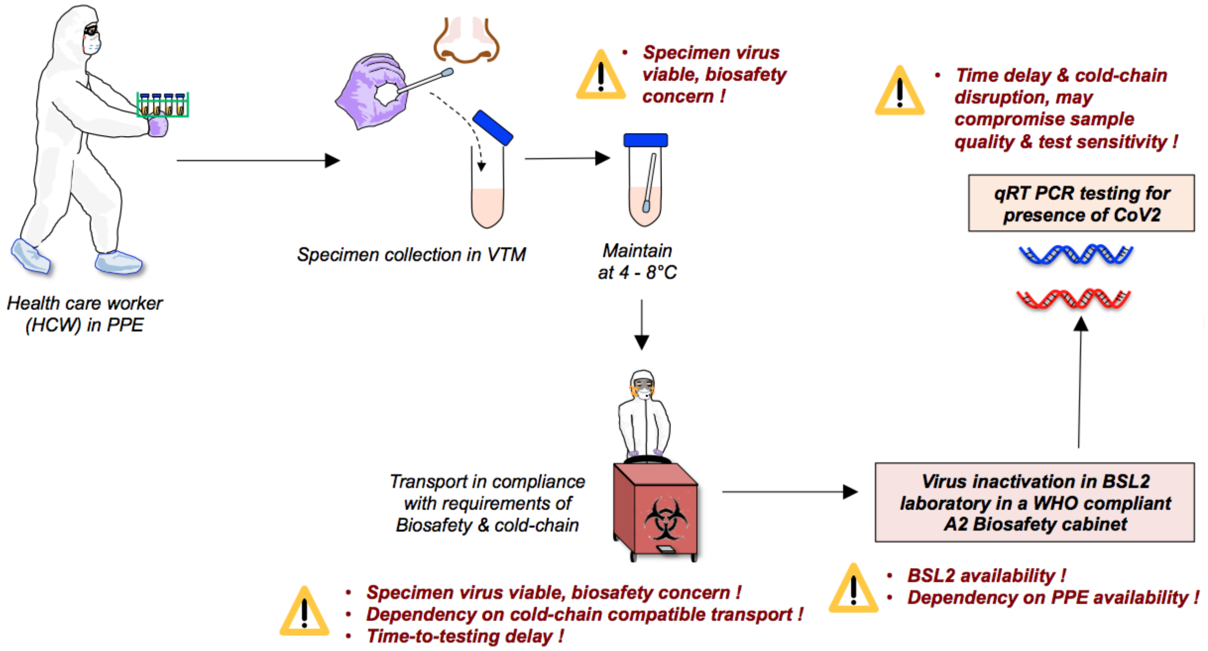

Supplemental Figure S2 M13KE bacteriophage recovery in MTM solution A versus PBS at various time-points.

Data represents recovery of M13KE phage when treated with PBS or Solution A (later named as SupraSens MTM or SSTM). Untreated sample was used as control. Data represents mean ± standard deviation of obtained PFU/mL. Experiments were performed at least twice in duplicates and analyzed using two way ANOVA Tukey's multiple comparison test (***p < 0.0001).

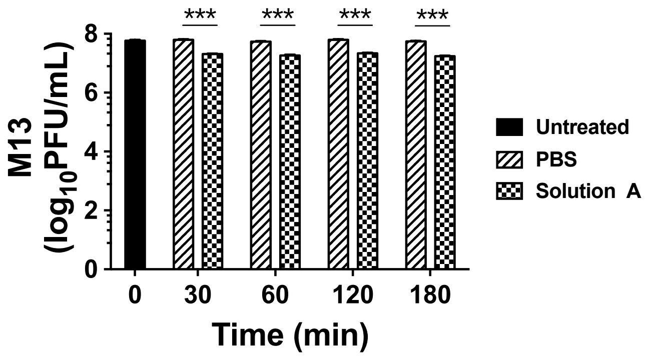

Supplemental Figure S3 Schematic of various approaches adopted to determine the clinical suitability of SSTM and evaluate its performance in Covid19 qRT PCR testing.

A: In approach 1, we determined the compatibility of SSTM with the existing clinical workflow of Covid19 testing using clinical specimens (N=30) stored in VTM. Samples were processed in parallel in exactly similar fashion except that one of them was processed using SSTM followed by RNA isolation and qPCR testing. **B:** In approach 2, we performed a simultaneous collection of clinical specimens (N=181) from same individual in SSTM and VTM, followed by processing, to compare qRT PCR performances. SSTM sample collection was at field temperature and transport did not have any cold chain or specific packaging requirements.

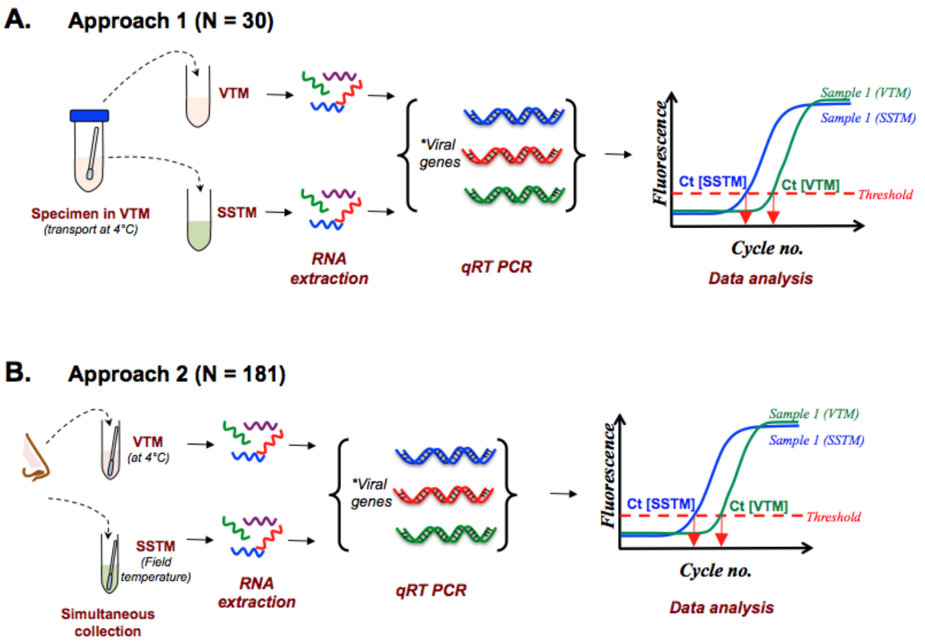

Supplemental Figure S4 ROC curve analysis of qPCR data in binary form reveals loss of sensitivity of Covid19 detection in samples processed in VTM.

ROC curves were generated after converting the qPCR Ct values of **A:** N gene, **B:** ORF1ab, **C:** S gene to binary format such that Ct value <37 was given code = 1 (for gene present); and Ct value >37 was given code = 0 (for gene absent). Specimens with qPCR signals 'not detected' due to low/no viral load were assigned code = 0. ROC curves of respective panels were compared as VTM vs SSTM using 2-sample Z test (***p < 0.0001) to analyze loss of diagnostic efficiency. [Note: TaqPath^{TM} COVID19 Combo kit defines a Ct cut-off value of <37 for Covid19 disease.]

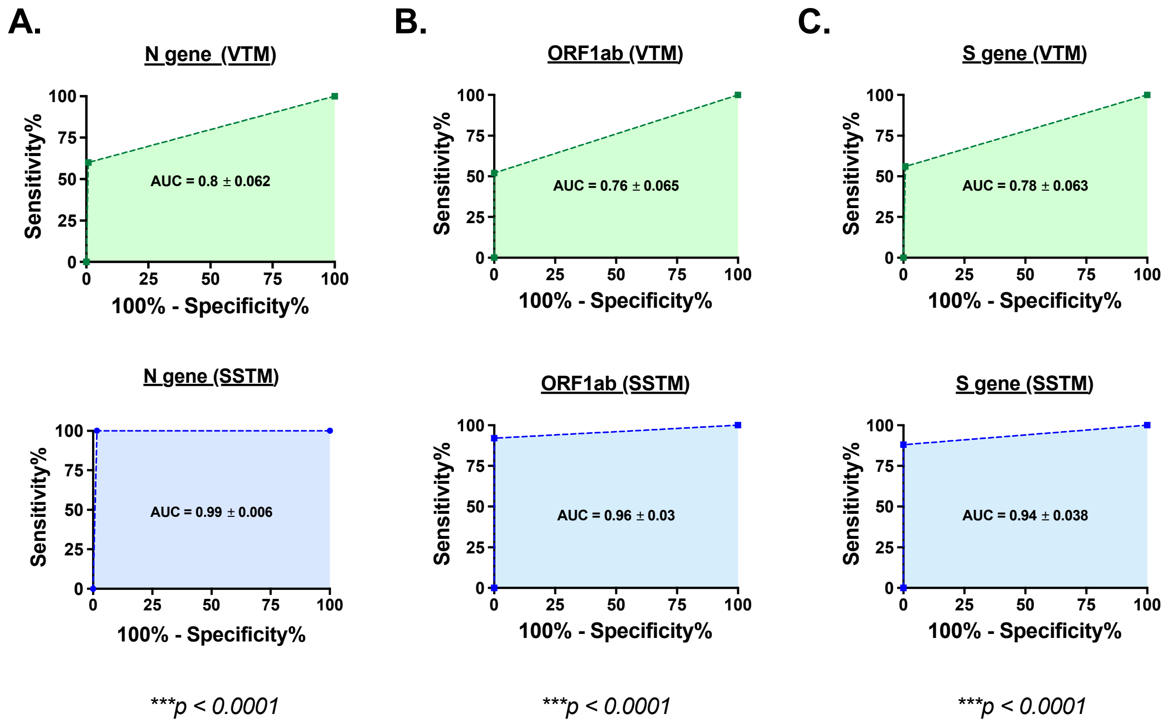

SUPPLEMENTAL TABLES

Supplemental Table S1 Clinical status, parameters and diagnostic findings in study participants.

S.No.	Covid-19 qPCR kit	Individuals enrolled	Median age	Gender distribution	Symptoms	Test positivity (by VTM)	Test positivity (by SSTM)
1	TaqPath™ COVID-19 Combo kit (Thermo Fischer Scientific)	149	38	33 (Females)<br>116 (Males)	51 (Symptomatic)<br>98 (Asymptomatic)	10 (Symptomatic)<br>4 (Asymptomatic)	14 (Symptomatic)<br>11 (Asymptomatic)
2	TRUPCR® SARS-CoV-2 RT qPCR kit (3B Blackbio Biotech India Ltd)	32	42	6 (Females)<br>26 (Males)	6 (Symptomatic)<br>26 (Asymptomatic)	1 (Symptomatic)<br>5 (Asymptomatic)	1 (Symptomatic)<br>8 (Asymptomatic)
**Total**		181	39	39 (Females)<br>142 (males)	57 (Symptomatic)<br>124 (Asymptomatic)	11 (Symptomatic)<br>9 (Asymptomatic)	15 (Symptomatic)<br>19 (Asymptomatic)

Supplemental Table S2 Comparative loss of Covid19 diagnostic test performance[#] in clinical samples processed in VTM vs SSTM.

		VTM method [Patients = 9; Controls = 23]				SSTM method [Patients = 9; Controls = 23]				% Loss in VTM sensitivity*	Z-test comparing Sensitivity% of VTM vs SSTM [$]	
		**Sensitivity[†] %**	**Specificity[‡] %**	**NPV[‖] %**	**PPV[¶] %**	**Sensitivity %**	**Specificity %**	**NPV %**	**PPV %**		**Z-score**	**P value**
**TRUPCR® SARS-CoV-2 RT qPCR kit**	**E gene**	66.7 [35.4 - 87.9] [§]	100 [85.7 - 100]	88.5 [71 - 96]	100 [61 - 100]	88.9 [56.5 - 98]	100 [85.7 - 100]	95.8 [79.8 - 99.3]	100 [67.6 - 100]	**22.2**	2.1	0.0326
	**RdRP+ N gene**	55.6 [35.4 - 87.9]	100 [85.7 - 100]	85.2 [67.5 – 94.1]	100 [56.6 - 100]	100 [70.1 - 100]	100 [85.7 - 100]	100 [85.7 - 100]	100 [70.1 - 100]	**44.4**	4.3	<0.0001
**Overall kit result**		66.7 [45.3 - 93.7]	100 [85.7 - 100]	88.5 [71 - 96]	100 [61 - 100]	100 [70.1 - 100]	100 [85.7 - 100]	100 [85.7 - 100]	100 [70.1 - 100]	**33.3**	3.6	0.0003

[#] Covid19 diagnostic test performed on N=32 samples using TRUPCR® SARS-CoV-2 RT qPCR kit (3B Blackbio Biotech India Ltd). Diagnostic test performance evaluated using online tool https://ebm-tools.knowledgetranslation.net/calculator/diagnostic [1,2]

* %Loss in VTM sensitivity = %Sensitivity in SSTM - %Sensitivity in VTM

[$] **Z test** for comparison of sensitivities between VTM versus SSTM using online tool https://epitools.ausvet.com.au/ztesttwo [3,4]

† Sensitivity% = [True positive / (True positive + False negative)] × 100

‡ Specificity% = [True negative / (True negative + False positive)] × 100

‖ Negative Predictive Value (NPV)% = [True negative / (True negative + False negative)] × 100

¶ Positive Predictive Value (PPV)% = [True positive / (True positive + False positive)] × 100

§ Values in parentheses denote 95% confidence interval

REFRENCES

1. Litvin TV, Bresnick GH, Cuadros JA, Selvin S, Kanai K, Ozawa GY: A revised approach for the detection of sight-threatening diabetic macular edema. JAMA Ophthalmol 2017, 135(1):62. doi:10.1001/jamaophthalmol.2016.4772
2. Bolboacă SD: Medical Diagnostic Tests: A review of test anatomy, phases, and statistical treatment of data. Comput Math Methods Med 2019, 2019:1891569. doi:10.1155/2019/1891569
3. Frazier TW, Coury DL, Sohl K, Wagner KE, Uhlig R, Hicks SD, Middleton FA: Evidence-based use of scalable biomarkers to increase diagnostic efficiency and decrease the lifetime costs of autism. Autism Research 2021, 14(6):1271-1283. doi:10.1002/aur.2498
4. Kourouche S, Curtis K, Munroe B, Asha SE, Carey I, Considine J, Fry M, Lyons J, Middleton S, Mitchell R, Shaban RZ, Unsworth A, Buckley T: Implementation of a hospital-wide multidisciplinary blunt chest injury care bundle (ChIP): Fidelity of delivery evaluation. Aust Crit Care 2021, S1036-7314(21)00058-8. doi:10.1016/j.aucc.2021.04.003
